# Antioxidant and gastric cytoprotective prostaglandins properties of *Cassia sieberiana* roots bark extract as an anti-ulcerogenic agent

**DOI:** 10.1186/1472-6882-12-65

**Published:** 2012-05-20

**Authors:** Edmund T Nartey, Mark Ofosuhene, William Kudzi, Caleb M Agbale

**Affiliations:** 1 Centre for Tropical Clinical Pharmacology and Therapeutics, University of Ghana Medical School, P.O. Box GP 4236, Accra, Ghana; 2 Department of Clinical Pathology, Noguchi Memorial Institute for Medical Research, University of Ghana, P. O. Box LG 581, Legon, Ghana; 3 Department of Biochemistry, School of Biological Sciences, University of Cape Coast, Cape Coast, Ghana

## Abstract

**Background:**

*Cassia sieberiana* is a savannah tree with a wide phytotherapeutic application including the use of its roots in the management of various stomach disorders including gastric ulcer, stomach pains and indigestion. The aim of the study is to evaluate the antioxidant, gastric cytoprotective prostaglandins, secretory phospholipase A_2_, phytochemical and acute toxicity properties of *Cassia sieberiana* roots bark extract in a bid to justify its phytotherapeutic applications in gastric ulcer.

**Methods:**

Antioxidant and radical scavenging activities of the roots bark extract of *Cassia sieberiana* were assayed. Serum secretory phospholipase A_2_ (sPLA_2_) concentration and activity and the formation of gastric mucosal prostaglandins E_2_ (PGE_2_) and I_2_ (PGI_2_) were also assessed. Comparisons between means were performed using analysis of variance (ANOVA) followed by Students Standard Newman-Keuls *post hoc* analysis to determine statistical significance. P < 0.05 was considered significant.

**Results:**

The extract was found to possess significant ferric reducing antioxidant power and can scavenge hydroxyl radicals. The extract also possesses DPPH scavenging activity, can chelate ferrous ion and a dose-dependent protective effect against lipid peroxidation and free radical generation. Prostaglandin studies showed that the roots bark extract dose dependently increased gastric mucosal PGE_2_ and PGI_2_ levels and also decreased serum sPLA_2_ activity. Phytochemical analyses suggest that the roots extract contains polyhydroxyl/phenolic substances. Acute toxicity test showed no sign of toxicity up to a dose level of 2000 mg/kg body weight p.o.

**Conclusions:**

*C. sieberiana* roots extract possesses significant antioxidant and gastric cytoprotective prostaglandin properties as well as serum secretory phospholipase A_2_ inhibitory activity which could be due to its content of polyhydroxy and/or phenolic substances. This may justify its use as an anti-ulcerogenic agent in traditional medicine in West Africa.

## Background

*Cassia sieberiana* is a savannah tree with a wide application including the ethnopharmacological use of its roots in the management of various stomach disorders including gastric ulcer, stomach pains and indigestion [1]. At the Centre for Scientific Research into Plant Medicine (CSRPM) in Ghana, an aqueous suspension of the powdered roots bark is used to manage abdominal colic and pains associated with the joints. Earlier studies we conducted indicated that the aqueous roots bark extract of *C. sieberiana* possesses anti-ulcerogenic properties against gastric ulcers induced by various methods [2].

Among the various factors implicated in the pathophysiology of gastric ulcer disease are the roles played by endogenous generation of prostaglandins (PGs) and free radicals [3]. Free radicals play fundamental roles in many physiological reactions. Due to their high reactivity, they have been implicated in the causation of some degenerative diseases including stress, NSAID and *H. pylori* induced gastric ulcers [4,5]. They have been shown to mediate the micro-vascular disturbance that precedes stress, NSAID or ischaemic reperfusion induced gastric mucosal injuries and also in the pylorus ligation model of gastric ulcers [5,6]. In addition studies have shown that certain anti-oxidants also prevent gastric ulcers [7-9].

PGs, a family of lipid compounds derived from arachidonic acid pathway mediate a wide range of physiological functions including modulation of inflammation and gastrointestinal mucosal defence. Superficial injury to the gastric mucosa is known to trigger an acute inflammatory response, characterized by the increase in blood flow, as well as by plasma exudation and recruitment into the mucosa of leukocytes [10]. While the acute inflammatory response is aimed at reducing mucosal injury, there are circumstances in which this response can be dysregulated and can contribute to mucosal injury [11]. The major prostaglandins produced by human and rodent gastric mucosa are PGE_2_ and PGI_2_ and these PGs accelerate ulcer healing in both experimental animal models and in humans [12,13]. The dual contribution of PGs to inflammation and mucosal defence present a challenge to the development of anti-inflammatory drugs.

Phospholipids also play an important role in the preservation of gastrointestinal homeostasis [14]. Phospholipase A_2_ (PLA_2_), an enzyme is capable of hydrolyzing membrane phospholipids, which in the presence of high gastric acidity leads to mucosal damage. PLA_2_ mediated hydrolysis of membrane lipids results in membrane perturbation, cell degranulation and stripping of cell surface receptors resulting in gastric ulcer. High concentrations of PLA_2_ have been reported in gastric mucosa [15] and PLA_2_ inhibitors are known to modulate proton conductance across cell membranes [16] and thus can offer gastric mucus protection from enzymatic breakdown. As a result of our earlier study on the phytotherapeutic ability of the roots bark of *C. sieberiana* as an anti-ulcerogenic agent, this study was conducted to assess the anti-oxidative (*in vitro*) and gastric mucosal prostaglandin properties of the roots bark extract of the plant.

## Methods

### Preparation of roots bark extract

Fresh roots of *C. sieberiana* (*Cassia kotschyana Oliv*.) were obtained from the grounds of the University of Ghana. The plant was identified and authenticated at the Ghana Herbarium, University of Ghana, Ghana. The chopped fresh roots bark (750 g) were grounded and mixed with 1 L of water and left to stand overnight. The resulting concoction was evaporated under reduced pressure at 30°C and the concentrate freeze-dried to yield solid material. The freeze-dried roots bark extract was stored at 4°C and used within 4 weeks of production.

### Animals

Male guinea pigs weighing 318 ± 30.3 g (mean ± S.D) were used for the prostaglandin studies. They were feed mainly on the leaves of *Panicum maximum* (Elephant grass) supplemented with standard laboratory diet (GAFCO, Tema, Ghana). Fisher 344 (F_344_) rats weighing 230.3 ± 14.5 g (mean ± S.D) of both sexes were used for the acute oral toxicity studies. They were also raised on standard laboratory diet (GAFCO, Tema, Ghana). The animal experimentation described in this study was approved by the Ethical and Protocol Review Committee of Noguchi Memorial Institute for Medical Research of the University of Ghana and was conducted in accordance with internationally accepted principles for laboratory animal use and care.

### Chemicals and reagents

L-ascorbic acid (LAA) was obtained from Sigma Chemical Company, St. Loius, U.S.A. All of the reagents for the antioxidant studies were obtained from Fluka Chemie, Switzerland. The enzyme immunoassay kits for prostaglandin E_2_ (Cat # 514010) and prostaglandin I_2_ (assayed as 6-keto PGF_1α_, Cat # 515211) as well as secretory phospholipase A_2_ (human Type IIa, Cat # 585000) and secretory phospholipase A_2_ activity (Type IIa, Cat # 765001) were obtained from Cayman Chemical Company (Am Arbor, U.S.A.). Arachidonic acid, reduced glutathione (GSH) and bovine serum albumin (BSA) were obtained from Sigma Chemical Company (St. Louis, MO, U.S.A.).

### *In vitro* antioxidant studies

All of the reagents for the antioxidant studies were prepared in deionised water to eliminate the contamination of metal ions. Total phenolic concentrations were determined as described by AOAC [17]. Total reducing power of the plant extract was determined by the method of Yildirim et al. [18]. DPPH radical scavenging activity was determined by the method of Zhu et al. [19]. Antioxidant activity in a haemoglobin-induced linoleic acid system of the roots extract was determined by a modified photometry method as described by Kuo et al. [20]. The hydroxyl scavenging activity was measured by studying the competition between deoxyribose and the extract for hydroxyl radicals generated from the Fe^3+^/ ascorbate/ EDTA/ H_2_O_2_ system [21]. Ferrous ion chelating effect was determined according to the method of Dinis et al. [22]. These assays were done in triplicate and L-ascorbic acid (LAA) was used as positive control.

### Gastric prostaglandins studies

#### Administration of plant extract

Different concentrations of the roots extract 250 mg/kg, 500 mg/kg and 750 mg/kg body weight, suspended in water (as vehicle) in a total volume of 2.0 mL were administered orally to three groups of guinea pigs made up of 5 animals per group. Treatment was repeated every 24 hours for 28 consecutive days. The control group received the vehicle instead of the roots extract.

#### Blood sampling

A day after the administration of the last dose of the plant extract, each animal was anaesthetised using sodium pentobarbitone (60 mg/kg body weight, i.p). A total volume of 5 mL of blood was obtained from each anaesthetised animal by cardiac puncture and a sample of 100 μL is diluted 1 in 9 using pre-chilled 25 mM Tris–HCl. This portion was used for the determination of serum secretory phospolipase A_2_ (sPLA_2_) concentration. A different portion of the obtained blood was allowed to clot and the serum used to estimate sPLA_2_ activity and protein content. The protein content in serum was determined by the Folin-Lowry method [23].

#### Eicosanoid biosynthesis and assay

Briefly after euthanizing the animals, the stomach were rinsed in pre-chilled 0.15 M KCl and then homogenised using 0.25 M sucrose-10 mM Tris–HCl (pH 7.5). The homogenate was centrifuged at 10,000 g for 20 min at 4°C and the supernatant centrifuged again at 105,000 g for 60 min at 4°C. The pellet was homogenised in 0.1 M K_2_PO_4_ buffer (pH 7.6). The eicosanoids prostaglandin E_2_ (PGE_2_) and prostaglandin I_2_ (PGI_2_) (assayed as 6-keto PGF_1α_) were biosynthesised using GSH as co-factor in the cyclooxygenase reaction. Reactions were terminated after 5 minutes using 100 μL of 1.0 M citric acid and assayed with ELISA kit from Cayman Chemical Company (Am Arbor, U.S.A.).

#### Secretory phospholipase A_2_ concentration and activity

Secretory phospholipase A_2_ concentration and secretory phospholipase A_2_ activity in the serum were assayed using kits (Cat # 585000 and Cat # 765001 respectively) from Cayman Chemical Company (Am Arbor, U.S.A.).

#### Phytochemical analyses

Standard phyto-chemical tests were used for the detection of tannins, saponins, flavonoids and anthraquinones in the extract [24]. Thin layer chromatography was also conducted on the plant extract using different mobile phase systems and silica gel 60F_254_ as the stationary phase. The chromatograms developed were viewed under daylight, UV light and vaporised iodine. The resolved components under UV light were treated with 0.1 mM FeCl_3_.

#### Acute toxicity

Acute oral toxicity study was performed as per OECD-423 guidelines (acute oral toxicity-acute toxic class method). Specific pathogen free F_344_ rats of either sex selected by random sampling technique were fasted overnight with water *ad libitum* after which the extracts (dissolved in water) were administered orally by gastric instillation beginning from a dose of 5 mg/kg body weight. The rats were observed for death within 48 hours after extract administration and surviving animals observed for 14 days for weight changes, lethargy and behavioural modifications. If mortality was not observed, the procedure was repeated with higher doses such as 50, 300 and 600 up to 2000 mg/kg body weight.

#### Statistical analysis

Comparisons between means were performed using analysis of variance (ANOVA) followed by Students Standard Newman-Keuls *post hoc* analysis to determine statistical significance. P < 0.05 was considered significant.

## Results

### Antioxidant studies

#### Amount of total phenolic compounds

Analyses of an amount of 200 μg of freeze-dried extract of *C. sieberiana* yielded 75.32 ± 0.52 of gallic acid equivalents of polyphenols. Therefore total phenolic compounds constituted 37.66 ± 0.27% of *C. sieberiana* roots bark extract.

#### Reducing power

**Figure 1:**
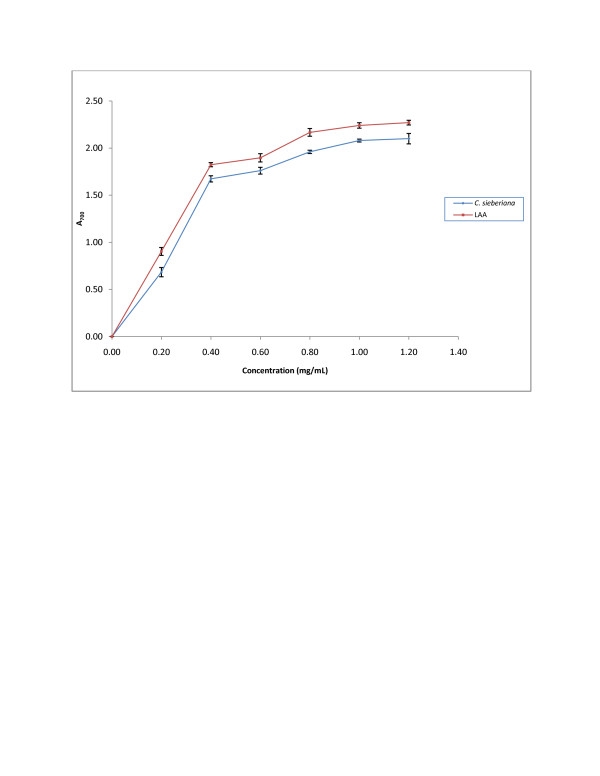
**Reducing power of*C. sieberiana*roots bark extract.** L-ascorbic acid (LAA) was used as a reference compound. Results are means ± SEM of n = 3.

The reducing power of the roots extract of *C. sieberiana* and LAA as reference compound are shown in Figure1. The graph shows that the reducing power increased with increasing concentration of *C. sieberiana* or LAA up to a concentration of 0.8 mg/mL after which there was no further increase in reducing power with increasing concentration of *C. sieberiana* extract or LAA. Analysis of the data indicated that below a concentration of 0.6 mg/mL for both *C. sieberiana* and LAA, significantly higher concentration of *C. sieberiana* than LAA was required to produce the same absorbance. The concentrations to attain one absorbance unit at 700 nm were 0.28 ± 0.03 mg/mL for *C. sieberiana* and 0.26 ± 0.02 mg/mL for LAA.

#### DPPH radical scavenging activity

**Figure 2:**
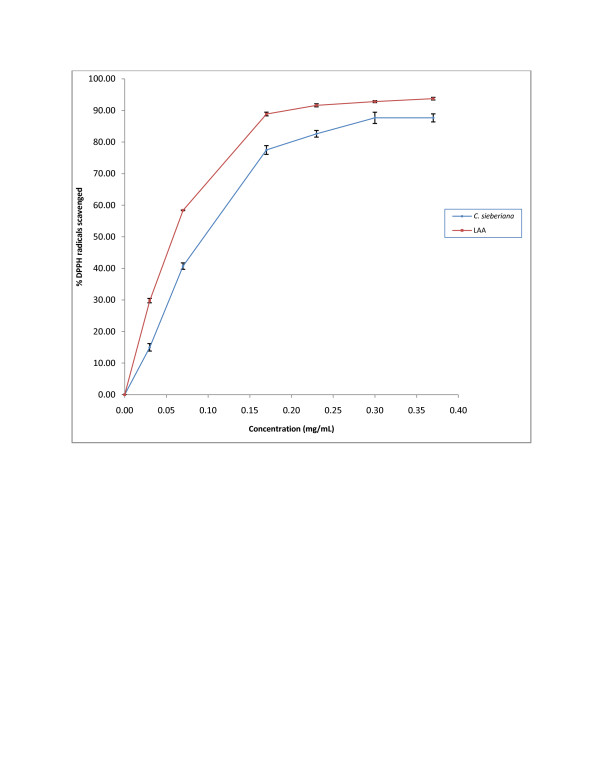
**DPPH radical scavenging activity of*C. sieberiana*roots bark extract.** L-ascorbic acid (LAA) was used as a reference compound. Results are means ± SEM of n = 3.

In the current study the scavenging activities of DPPH exerted by *C. sieberiana* and LAA shows a linear response curve (Figure2) and the IC_50_ estimated for *C. sieberiana* and LAA were 0.095 ± 0.006 mg/mL and 0.06 ± 0.004 mg/mL respectively. This significantly different IC_50_ value of the crude extract was however 63.2% that of LAA.

#### Scavenging effect on hydroxyl radical

**Figure 3:**
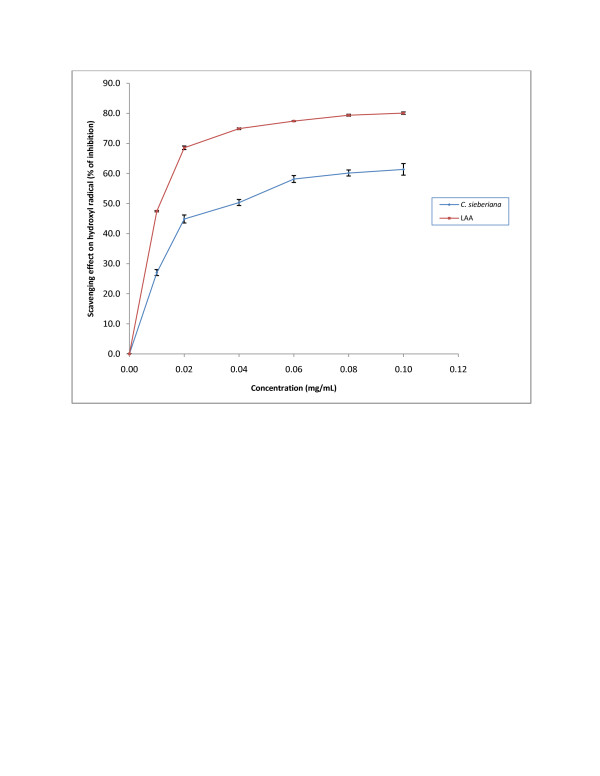
**Hydroxyl radical scavenging activity of*C. sieberiana*roots bark extract.** L-ascorbic acid (LAA) was used as a reference compound. Results are means ± SEM of n = 3.

In this study, the roots extract was able to achieve a 62% maximum scavenging activity when its concentration was more than 10 mg/mL (Figure3). Nevertheless, LAA had about 70% scavenging activity at the low concentration of 0.02 mg/mL. The IC_50_ estimated for *C. sieberiana* was 0.04 ± 0.006 mg/mL whilst that for LAA was 0.01 ± 0.002 mg/mL.

#### Antioxidant activity in a haemoglobin-induced linoleic acid system

**Figure 4:**
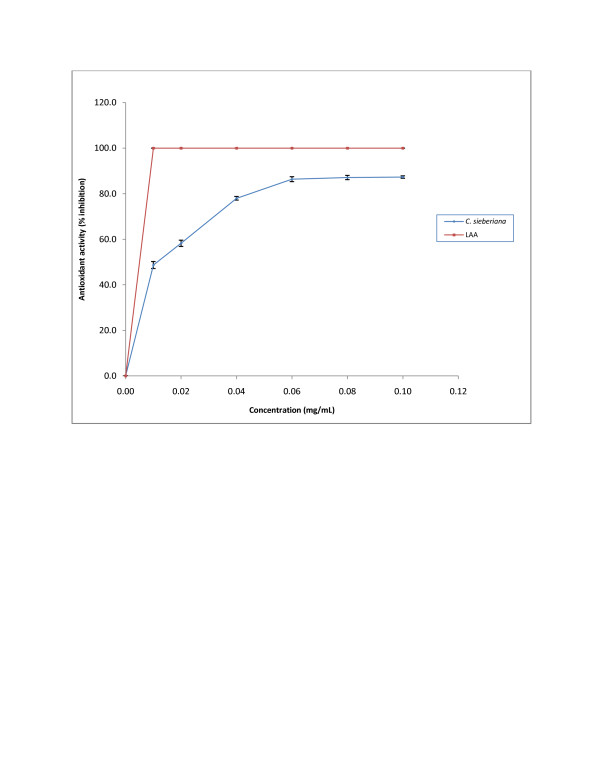
**Antioxidant activity of*C. sieberiana*roots bark extract against linoleic acid peroxidation induced by haemoglobin.** L-ascorbic acid (LAA) was used as a reference compound. Results are means ± SEM of n = 3.

As shown in Figure4 the antioxidant activity was dose dependent and reached a plateau (about 77-87% inhibition) when the concentration of *C. sieberiana* exceeded 0.06 mg/mL. This result indicates that the antioxidant activity of *C sieberiana* is only about 17% that of LAA as shown by the peroxidation in the presence of 1 mM linoleic acid. The IC_50_ estimated for *C. sieberiana* was 0.012 ± 0.004 mg/mL and that for LAA was 0.006 ± 0.002 mg/mL.

#### Ferrous ion chelating effect

**Figure 5:**
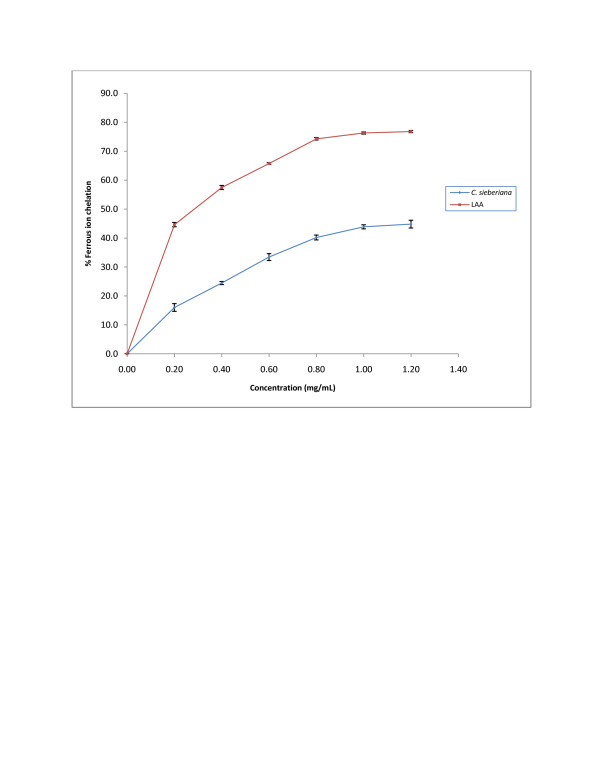
**Ferrous ion chelating effect of*C. sieberiana*roots bark extract.** L-ascorbic acid (LAA) was used as a reference compound. Results are means ± SEM of n = 3.

The ferrous ion chelating activity of the *C. sieberiana* roots extract shows a linear response curve and graph analysis shows IC_50_ estimation was 3.20 ± 0.24 mg/mL for *C. sieberiana* and 0.28 ± 0.024 mg/mL for LAA (Figure5).

#### PGE_2_, PGI_2_ and sPLA_2_ studies

Daily administration of the roots extract for 28 days produced a dose dependent increase in the amounts of gastric mucosal PGE_2_ and 6-keto PGF_1α_ synthesised (Table 1). Compared with the control, PGE_2_ increased by 37.7% in the extract low dose (250 mg/kg body weight), 64.7% in the extract medium dose (500 mg/kg body weight) and 82.4% in the extract high dose (750 mg/kg body weight). All the doses of the roots extract produced significant increases in 6-keto PGF_1α_ by 28.3% in the low dose, 61.7% in the medium dose and 88.6% in the high dose.

**Table 1:** **Effect of roots bark extract of*C. sieberiana*on mucosal PGE**_**2**_**and 6-keto PGF**_**1α**_**levels and serum sPLA**_**2**_**activity in guinea pigs**

**Treatment**	**Dose (mg/kg)**	**PGE**_**2**_**(pg/mL) (Mean ± S.E.M)**	**6-keto PGF**_**1α**_**(pg/mL) (Mean ± S.E.M)**	**sPLA**_**2**_**activity (nmol/min/mL) (Mean ± S.E.M)**
Control	vehicle	0.17 ± 0.02	2.10 ± 0.19	26.44 ± 0.69
Extract	250	0.23 ± 0.01^*^	2.69 ± 0.08^*^	20.84 ± 0.65^‡^
“	500	0.28 ± 0.02^†^	3.39 ± 0.34^*^	18.16 ± 0.68^‡^
“	750	0.31 ± 0.02^‡^	3.95 ± 0.19^‡^	10.52 ± 0.52^‡^

^*^ p < 0.05, statistically significant relative to control.

^†^ p < 0.01, statistically significant relative to control.

^‡^ p < 0.001, statistically significant relative to control.

Oral administration of the roots extract also produced a significant inhibition of serum sPLA_2_ activity but no significant change in the amount of serum sPLA_2_ protein. Compared with the control, the low dose produced 21.2% decrease, whilst the medium dose and the high dose produced 31.3% and 60.2% decrease in serum sPLA_2_ activity respectively.

#### Phytochemical screening

Phytochemical screening revealed the presence of saponins, flavonoids, anthraquinones and tannins in the roots extract. Thin layer chromatography revealed by their reaction with FeCl_3_ and their fluorescence under UV-light indicated that the roots extract contains flavonol/flavonoid/flavone or related compounds with polyhydroxy and/or phenolic groups.

#### Acute toxicity

In F_344_ rats of either sex, the extracts (5–2000 mg/kg, p.o) given as a single dose did not produce any signs of acute toxicity. No mortality was observed during the study period of 14 days and at necropsy, no gross changes in viscera were observed in the treated groups.

## Discussion

The present study was undertaken to investigate the antioxidant and gastric cytoprotective prostaglandin properties of the aqueous roots extract of *C. sieberiana*, which we had previously demonstrated to possess anti-ulcerogenic properties. Gastric ulcer disease is a multi-factorial disease and among the various properties implicated in its pathophysiology is the role played by free radicals. Hence antioxidants which can scavenge free radicals are expected to heal or prevent gastric ulcers. Several methods developed to measure the efficiency of antioxidants focus on different mechanisms of the oxidant defence system. In most of the systems, irrespective of the stage in the oxidative chain in which the antioxidant action is measured, most non-enzymatic anti-oxidative activity is mediated by redox reactions. The redox potentials of compounds are related to their antioxidant activity against free radicals such as peroxyl or hydroxyl radicals, which have more positive redox potentials [25]. As shown in Figure1 the reducing power increased as the extract concentration increased indicating some compounds in the roots extract were both electron donors and could react with free radicals to converts them into more stable products to terminate radical chain reactions. This result indicates that although the reducing power of LAA was slightly higher it was not significantly different from that of *C. sieberiana* (p > 0.05). The significant reducing powers recoded therefore suggested that the roots extract of *C. sieberiana* would have significant antioxidant action which was subsequently confirmed by its scavenging action of free radicals generated by DPPH.

In solution, DPPH generates free radicals whose odd electrons become paired off in the presence of a hydrogen donor. The DPPH radical has been widely used to test the free radicals scavenging ability of various natural products and has been accepted as a model compound for free radicals originating in lipids [26]. In DPPH assay the lower the IC_50_, the better it is able to scavenge the radicals, particularly peroxy radicals which are the propagators of the auto-oxidation of lipid molecules and thereby break the free radical chain reaction. Like the reducing power, at the IC_50_ of 0.075 ± 0.006 mg/mL, the DPPH scavenging activity of the crude extract was 63.2% that of LAA which indicates that the roots extract contains compounds that have strong antioxidant properties. Hydroxyl radical is the principal contributor for tissue injury. The deoxyribose method is used in determining the rate constants of reactions involving hydroxyl radicals. The reaction involves the incubation of a mixture of FeCl_3_-EDTA, H_2_O_2_ and ascorbate with deoxyribose in phosphate buffer (pH 7.4). The hydroxyl radicals generated from the mixture attack the deoxyribose and result in a series of reactions that cause the formation of MDA. A hydroxyl radical scavenger added to the reaction mixture will therefore compete with deoxyribose for the availability of hydroxyl radicals, thereby reducing the amount of MDA formed. In this study, the roots extract of *C. sieberiana* was able to achieve a 62% maximum scavenging activity indicating that it contains compounds that inhibit hydroxyl scavenging activity.

Lipid peroxidation has been implicated in the pathogenesis of various diseases including gastric ulcer disease in both humans [27] and experimental animals [28]. It is well established that bio-enzymes are very much susceptible to lipid peroxides, which is considered to be the starting point of many degenerative processes. This result indicates that the antioxidant activity of *C sieberiana* is only about 17% that of LAA as shown by the peroxidation in the presence of 1 mM linoleic acid which was significantly lower compared with LAA. The chelating ability of the extract measures how effective the compounds in it can compete with ferrozine for Fe^2+^. The Fe^2+^-ferrozine complex has maximum absorbance at 562 nm and a large decrease in absorbance indicates strong chelating power. By forming a stable Fe^2+^ chelate, an extract with high chelating power reduces the free Fe^2+^ concentration thus decreasing the extent of Fenton reaction which is implicated in many diseases. The results indicate that the crude roots extract can chelate free Fe^2+^.

The observed antioxidant activity is significant because free radicals have been implicated to mediate in stress, NSAID and *H. Pylori* induced gastric ulcers [5,29]. Neutrophil adherence to the endothelium of gastric microcirculation has also been shown to be critical in mucosal injury in animals and such adherence is believed to liberate oxygen radicals, resulting in the release of proteases and obstructing capillary blood flow or causing lipid peroxidation and damaging cell membranes [30]. Therefore the reported anti-ulcer activity of the roots extract of *C. sieberiana* could be due in part to its strong reducing and antioxidant properties.

Phytochemical analysis detected alkaloids, saponins, anthraquinones, tannins and flavonoids in the *C. sieberiana* roots extract. TLC spots revealed by their reaction with ferric chloride and their fluorescence under UV light indicated that flavonol/flavonoid/flavone or related compounds with polyhydroxy and/or phenolic groups constituted major chemical substances in the roots extract. Evidence of the presence of flavonol/flavonoid/flavone or a related compound with polyhydroxy and/or phenolic groups is consistent with the significant antioxidant effect of the roots extract. Flavonoids or polyphenolic substances exert antioxidant actions by scavenging free radicals, chelating metal ions or inhibiting enzyme systems that generate free radicals. Several studies demonstrated that flavonoids from various plants are reportedly capable of preventing the occurrence of gastric ulcer. This may take place through an increase in the amounts of neutral glycoproteins and in prostaglandin concentrations, and inhibition of histamine secretion from mast cells by inhibition of histidine decarboxylase, thus reducing stimulation of H_2_ receptors, or by secretion of prostaglandin-like compounds [31]. Another possible mechanism of action for inhibiting ulcer occurrences is by decreasing pepsin secretion and activity. In addition, several studies cited by Middleton and Kandaswami [32], indicate that certain flavonoids have anti-ulcer activity with some having direct mucosal protection activity similar to that of prostaglandins. This suggests that flavonoids and/or polyphenols may be responsible for the reported gastric mucus protection and anti-ulcer properties of the roots bark extract of *C. sieberiana*.

The major prostaglandins produced by human and rodent gastric mucosa are PGE_2_ and PGI_2_ which are vasodilators in the gastrointestinal mucosa [11]. The vasodilatory properties of these two molecules may increase mucus production and reduce acid and pepsin levels in the stomach, thereby make an important contribution to gastric mucosal defence and facilitate the repair of pre-existing ulcers in the gastrointestinal mucosa [33,34]. The present results show that the significant dose-dependent increase in gastric mucosal PGE_2_ and PGI_2_ (82.4% and 88.6% respectively in the high dose) in the absence of induced gastric ulcers is an indicator of anti-ulcerogenic activity of the roots bark extract. These results confirm an earlier study [2] on the anti-ulcerogenic properties of the roots bark extract which showed that its gastric cytoprotective effect in rats was reduced significantly by pre-treatment with indomethacin, an NSAID prostaglandin inhibitor.

The inflammatory process which occurs during gastric ulceration is also known to be a key component of mucosal defence against exogenous and endogenous factors. However, while the acute inflammatory response is aimed at reducing mucosal injury, some of the inflammatory mediators released increase the susceptibility of the stomach to damage induced by NSAIDs or other topical irritants, and thereby contributes to the generation of mucosal injury in certain circumstances [11]. Several of these inflammatory mediators including histamine, tumor necrosis factor-α, platelet-activating factor, interleukin-1, interleukin-8 and leukotriene B_4_ have been shown to be down-regulated by endogenous generation of prostaglandins [11]. In addition prostaglandins are also potent inhibitors of leukocyte adherence to the vascular endothelium which is a hallmark of inflammation such that leukocyte adherence that occurs within the gastrointestinal microcirculation following administration of an NSAID can be prevented by prostaglandin administration [35,36]. It is imperative to note that activated inflammatory cells may also be potential sources of free radicals [37] which further aggravate the damaged gastric mucosa. This may be a potential mechanism of action of anti-oxidants as anti-ulcerogenic agents.

The roots extract did not show decline of sPLA_2_ protein; however, enzymatic activity of sPLA_2_ in serum was definitely inhibited. Thus, the impact may be on the enzyme-substrate level possibly by blunting sPLA_2_ interaction with its substrates rather than on the synthesis of sPLA_2_. It was expected that the stimulation of endogenous generation of gastric mucosal PGE_2_ and PGI_2_ will be coupled with an increase of arachidonic acid signaling as a result of stimulated sPLA_2_. The stimulation of endogenous generation of gastric mucosal PGE_2_ and PGI_2_ despite the inhibition of serum sPLA_2_ is interesting. An important clue may lie in the fact that the study measured sPLA_2_ in the serum and not in the stomach homogenate hence it is possible the extract is exerting its effect selectively at the two different locations due to the various isoforms of sPLA_2_ discovered [38,39]. However, the inhibition of sPLA_2_ in the serum confirms an earlier report which suggests that the inhibition of serum sPLA_2_ by *C. sieberiana* roots bark extract may be partly responsible for its therapeutic use as an anti-asthmatic and anti-inflammatory agent [40]. We are currently conducting studies into the effect of different fractionated portions of the extract on sPLA_2_ activity and expression in different tissues in a bid to elucidate its mechanism of action.

Despite these findings, inhibition of gastric mucosal sPLA_2_ activity may indicate anti-ulcerogenic abilities. Ischemic insult to intestinal mucosa causes excessive production of oxygen derived free radicals (ODFR) that may initiate a chain of reactions in membrane-bound lipids leading to lipid peroxidation and PLA_2_ activation [41,42]. Inhibition of this cascade by scavenging ODFR and/or inhibiting sPLA_2_ could therefore be an effective tool to protect gastrointestinal mucosa against chemically induced lesions.

## Conclusions

Antioxidant properties can be primary or secondary. Primary antioxidants scavenge radicals to inhibit chain initiation and break chain propagation. Secondary antioxidants suppress the formation of radicals and protect against oxidative damage by binding to metal ions strongly. Primary antioxidant properties are generally measured by DPPH assay and ferric reducing antioxidant power whilst secondary antioxidant properties is generally measured by the ferrous ion chelating activity which measures the chelating ability of the extract. In this study we demonstrated that roots bark extract of *C. sieberiana* have both DPPH scavenging activity and ferric reducing power and can scavenge hydroxyl radicals. The extract was also able to arrest lipid peroxidation and chelate ferrous ion. The roots extract therefore do possess both primary and secondary antioxidant properties which may be due to the high content of phenolic compounds. The roots extract was responsible for the observed stimulation of endogenous generation of gastric mucosal PGE_2_ and PGI_2_, and the inhibition of sPLA_2_ activity in serum. These findings suggest that the plant extract contains bimolecules which has high antioxidant properties and can stimulate the endogenous generation of gastric mucosal PGE_2_ and PGI_2_ which may be responsible for the therapeutic use as an anti-ulcer agent. The extract also showed no sign of acute toxicity at a dose of 2000 mg/kg body weight p.o. At present, it remains uncertain how the plant extract can inhibit serum sPLA_2_ but stimulate endogenous generation of gastric mucosal PGE_2_ and PGI_2_ and further studies are currently underway to clarify this by investigating the effect of different fractions of the extract on sPLA_2_ activity and expression in different tissues.

## Competing interests

The authors declare that they have no competing interests.

## Authors’ contributions

ETN and MO worked in the conception and the article final composition. WK and CMA contributed in the methods, in analysing results and revising it critically. All the authors read and approved the final manuscript.

## Pre-publication history

The pre-publication history for this paper can be accessed here:

http://www.biomedcentral.com/1472-6882/12/65/prepub
